# Neoadjuvant versus adjuvant chemotherapy in luminal B-like breast cancer: a subset analysis of the CANTO study

**DOI:** 10.1016/j.esmorw.2026.100789

**Published:** 2026-10-12

**Authors:** T. Gaillard, P. Cottu, O. Tredan, A.-L. Martin, C. Gaudin, A. Bertaut, C. Cheymol, C. Kaderbhai, F. Cherifi, M. Campone, M.-A. Mouret-Reynier, A. Di-Meglio, F. Reyal, A.S. Hamy-Petit, E. Laas

**Affiliations:** 1 Department of Surgical Oncology, Institut Curie, Paris, France; 2 Department of Medical Oncology, Institut Curie, Paris, France; 3 Institut Curie, Institut Hospitalo-Universitaire des Cancers des Femmes (ANR-23-IAHU-0006), Paris, France; 4 Université Paris Cité, Paris, France; 5 Unicancer Breast Cancer Group, Unicancer, Paris, France; 6 Department of Medical Oncology, Institut Léon Bérard, Lyon, France; 7 Data and Partnerships Department, UNICANCER, Paris, France; 8 Department of Methodology and Biostatistics, Centre Georges François Leclerc, Dijon, France; 9 Department of Medical Oncology, Centre Oscar Lambret, Lille, France; 10 Department of Medical Oncology, Centre Georges François Leclerc, Dijon, France; 11 Department of Medical Oncology, Centre François Baclesse, Caen, France; 12 Department of Medical Oncology, Institut de Cancérologie de L’ouest, Nantes Saint-Herblain, France; 13 Department of Medical Oncology, Centre Jean Perrin, Clermont-Ferrand, France; 14 Department of Medical Oncology, Cancer Survivorship Program, INSERM U981, Gustave Roussy, Villejuif, France; 15 Department of Surgical Oncology, Institut Godinot, Reims, France; 16 Residual Tumor & Response to Treatment Laboratory, RT2Lab, Translational Research Department, INSERM, U932 Immunity and Cancer, Institut Curie, Paris, France

**Keywords:** adjuvant chemotherapy, luminal B breast cancer, neoadjuvant chemotherapy

## Abstract

**Background:**

The benefit of neoadjuvant chemotherapy (NAC) in hormone receptor (HR)-positive/human epidermal growth factor receptor 2 (HER2)-negative breast cancer (BC) remains uncertain, given low pathologically documented complete response (pCR) rates and limited long-term comparative data. We compared overall survival (OS) between NAC and adjuvant chemotherapy (AC) in luminal B-like breast cancer treated in routine practice.

**Patients and methods:**

Patients enrolled in the prospective French CANTO cohort between January 2012 and December 2018 with unilateral, nonmetastatic, grade 3 HR-positive/HER2-negative BC (cT2–T3N0–N3M0) treated with NAC followed by surgery or with primary surgery followed by AC were included. The primary endpoint was OS; secondary endpoints were distant recurrence-free survival (DRFS) and invasive disease-free survival; exploratory endpoints were BC-specific survival, locoregional recurrence-free survival (LRFS) and breast recurrence-free survival. Inverse probability of treatment weighting (IPTW) was applied, with weighted Kaplan–Meier estimates and Cox models.

**Results:**

Among 544 patients, 165 received NAC and 379 AC; median follow-up was 72 months. Breast-conserving surgery was more frequent after NAC (64% compared with 45%, *P* < 0.001) and the pCR rate was 19%. After IPTW, the hazard ratio for OS with NAC was 1.36 [95% confidence interval (CI) 0.94-1.97, *P* = 0.10] univariably and 1.62 (95% CI 1.10-2.38, *P* = 0.014) after multivariable adjustment. No difference was observed for DRFS (hazard ratio 1.05, 95% CI 0.80-1.38, *P* = 0.70). LRFS was worse with NAC (hazard ratio 2.29, 95% CI 1.15-4.58, *P* = 0.018) in 19 events. In interaction tests, the association with OS was confined to cN0 (hazard ratio 2.39) and cT2 (hazard ratio 1.94) disease and absent in cN+ (hazard ratio 0.83) and cT3 (hazard ratio 0.88) tumors.

**Conclusions:**

NAC was associated with more locoregional recurrence without any difference in DRFS and the association with OS was confined to subgroups in whom a neoadjuvant indication is least established. These hypothesis-generating findings do not support the routine use of NAC in luminal B-like BC without an anticipated surgical benefit.

## Highlights

• Compared NAC versus AC in luminal B breast cancer using the CANTO cohort.
• NAC was linked to worse overall and locoregional survival after IPTW adjustment.
• No DRFS benefit was observed with NAC.
• NAC increased breast-conserving surgery rates despite low pCR rates.
• Findings support cautious use of NAC in HR-positive/HER2-negative grade 3 tumors.

## Introduction

Pathologically documented complete response (pCR), defined as the absence of residual invasive or noninvasive cancer in the breast and lymph nodes (ypT0-ypN0), is an established surrogate of improved survival after neoadjuvant chemotherapy (NAC),^1^, ^2^, ^3^, ^4^ and NAC has become the standard of care for most triple-negative breast cancer (TNBC) and human epidermal growth factor receptor 2 (HER2)-positive breast cancer (BC).^5^, ^6^, ^7^, ^8^, ^9^, ^10^, ^11^ Beyond increasing breast-conserving surgery, *in vivo* chemosensitivity testing allows postneoadjuvant treatment to be adapted to pathologically documented response (pathological response) in these subtypes, with a favorable impact on survival.^12^, ^13^, ^14^, ^15^

Luminal tumors are classified as A or B according to proliferation. Luminal A-like tumors (Ki67 <20%) respond poorly to chemotherapy, with pCR rates <5%, whereas luminal B-like tumors (Ki67 ≥20%) reach about 10%, compared with 21.6%-45% for estrogen receptor (ER)-negative tumors.^16^, ^17^, ^18^, ^19^ In the meta-analysis by Cortazar et al.^1^, pCR was not correlated with overall survival (OS) in ER-positive/HER2-negative grade 1/2 tumors [hazard ratio 0.47, 95% confidence interval (CI) 0.21-1.07], in contrast to grade 3 tumors (hazard ratio 0.29, 95% CI 0.13-0.65).

In luminal BC, NAC has been used mainly when breast-conserving surgery was not initially feasible or in the presence of nodal involvement. Given the modest pathological response of these tumors, comparing NAC and adjuvant chemotherapy (AC) is essential but methodologically difficult because patients selected for either strategy differ substantially and adjustment is required for a valid comparison.

Several registry-based analyses have addressed this question in HR-positive/HER2-negative disease, with inconsistent results. A Swedish population-based study using propensity score matching (1539 patients per group) found no difference in distant disease-free survival, breast cancer-specific survival (BCSS), or OS,^20^ whereas a Surveillance, Epidemiology, and End Results (SEER) database analysis of T2N1M0 tumors reported inferior survival after NAC, concentrated among patients without pCR.^21^ Most luminal A-like and B-like tumors were pooled and no locoregional endpoint was reported.

The aim of this study was to compare outcomes after NAC or AC in patients with luminal B-like BC within the multicenter prospective CANTO cohort,^22^ a population in which chemotherapy is generally indicated and the sequencing decision genuinely debated. The analysis is based on a prospectively assembled, centrally monitored cohort rather than an administrative registry and reports locoregional as well as distant endpoints.

## Methods

### Study design

CANTO is a prospective multicentric cohort study of treatment-related toxicities and long-term outcomes in nonmetastatic BC, coordinated by UNICANCER across 26 French cancer centers. Patients were enrolled between January 2012 and December 2018; the present analysis is based on the database lock in early 2023. All patients provided written informed consent and the study is registered at ClinicalTrials.gov (NCT01993498).^23^^,^^24^

We identified two groups of patients with luminal B-like cancers who underwent surgery and chemotherapy to compare outcomes of NAC followed by surgery with surgery followed by AC.

### Study population

CANTO required a histologically confirmed invasive BC staged cT0-cT3, cN0-3 (American Joint Committee on Cancer, seventh edition^25^), age ≥18 years, no prior treatment, including surgery, and written informed consent. Patients with stage T4 or IV disease, any prior local or distant recurrence, or another malignancy within 5 years were excluded.

For this subset analysis, the population was restricted to T2-3 N0-3 M0, invasive, grade 3, HR-positive/HER2-negative luminal B-like tumors; T2-T3 tumors were selected because NAC is not typically indicated for tumors <20 mm. Because Ki67 and progesterone receptor (PR) status were not available for all patients, histopathological grade 3 was used as a proxy for luminal B-like disease; tumors were designated ‘luminal B-like’ in accordance with the St Gallen convention, indicating a surrogate rather than an intrinsic definition. All patients underwent surgery for the primary tumor and those with inoperable (T4) or metastatic disease were not eligible for CANTO; a single patient (0.2%), in the NAC group, had cN3 disease.

### Study endpoints

The primary endpoint was OS, from diagnosis to death from any cause. Secondary endpoints were distant recurrence-free survival (DRFS), from diagnosis to distant recurrence or death, and invasive disease-free survival (IDFS), from diagnosis to locoregional or distant recurrence or death. Exploratory endpoints were BCSS, locoregional recurrence-free survival (LRFS), from diagnosis to recurrence in the breast or nodes, and breast recurrence-free survival (BRFS).

Patients without any event were censored at last follow-up. This study evaluates the comparative effectiveness of two treatment sequences as delivered in routine practice, not the efficacy of chemotherapy itself.

### Statistical analysis

Baseline characteristics were compared using the chi-square or Fisher’s exact one tailed test for categorical variables and the Wilcoxon rank-sum test for continuous variables, at a 5% significance level. The *P* values in Table 1 are descriptive and characterize treatment selection in routine practice; they were not used to assess balance. Propensity scores (PSs) were estimated by multivariable logistic regression.

**Table 1:** Characteristics according to chemotherapy strategy.

Variable	Overall *n* = 544	Chemotherapy		*P* value^a^
		NAC *n* = 165	AC *n* = 379	
Age	52 (24, 84)	51 (25, 71)	54 (24, 84)	0.001
Age range				0.030
<40	82 (15%)	35 (21%)	47 (12%)	
40-50	148 (27%)	41 (25%)	107 (28%)	
>50	314 (58%)	89 (54%)	225 (59%)	
Menopausal status				0.2
Postmenopausal	266 (49%)	74 (45%)	192 (51%)	
Premenopausal	275 (51%)	91 (55%)	184 (49%)	
(Missing)	3	0	3	
BMI	25.1 (16.4, 54.4)	24.4 (17.4, 54.4)	25.2 (16.4, 46.1)	0.6
BMI range				0.091
<18.5	8 (1.5%)	1 (0.6%)	7 (1.8%)	
18.5 to <25	263 (48%)	87 (53%)	176 (46%)	
25 to 30	150 (28%)	35 (21%)	115 (30%)	
≥30	123 (23%)	42 (25%)	81 (21%)	
Initial clinical tumor size				<0.001
T2	450 (83%)	119 (72%)	331 (87%)	
T3	94 (17%)	46 (28%)	48 (13%)	
Initial nodal status				<0.001
N0	356 (73%)	91 (61%)	265 (78%)	
N+	133 (27%)	58 (39%)	75 (22%)	
(Missing)	55	16	39	
Ki67	37 (1, 100)	42 (2, 100)	34 (1, 100)	0.002
(Missing)	135	54	81	
Histological type				0.002
NST	442 (81%)	147 (90%)	295 (78%)	
Lobular	41 (7.6%)	4 (2.4%)	37 (9.8%)	
Mixed types/other	60 (11%)	13 (7.9%)	47 (12%)	
(Missing)	1	1	0	
Tubule formation score (Elston–Ellis)				0.12
1	0 (0%)	0 (0%)	0 (0%)	
2	45 (10%)	9 (6.6%)	36 (12%)	
3	396 (90%)	127 (93%)	269 (88%)	
(Missing)	103	29	74	
Mitotic count score (Elston–Ellis)				<0.001
1	28 (5.2%)	15 (9.4%)	13 (3.4%)	
2	171 (32%)	65 (41%)	106 (28%)	
3	339 (63%)	79 (50%)	260 (69%)	
(Missing)	6	6	0	
Nuclear pleomorphism score (Elston–Ellis)				0.041
1	1 (0.2%)	1 (0.7%)	0 (0%)	
2	95 (21%)	22 (16%)	73 (24%)	
3	346 (78%)	114 (83%)	232 (76%)	
(Missing)	102	28	74	
ER (%)	95 (0, 100)	90 (0, 100)	95 (0, 100)	<0.001
(Missing)	22	4	18	
Progesterone receptor (%)	50 (0, 100)	30 (0, 100)	60 (0, 100)	<0.001
(Missing)	32	9	23	
ER (%)				<0.001
10%-80%	86 (16%)	46 (29%)	40 (11%)	
<10%	11 (2.1%)	8 (5%)	3 (0.8%)	
≥80%	425 (81%)	107 (66%)	318 (88%)	
(Missing)	22	4	18	
Progesterone receptor (%)				0.019
10%-80%	214 (42%)	64 (41%)	150 (42%)	
<10%	114 (22%)	46 (29%)	68 (19%)	
≥80%	184 (36%)	46 (29%)	138 (39%)	
(Missing)	32	9	23	
HER2 level				0.023
0	246 (45%)	89 (54%)	157 (42%)	
1	151 (28%)	36 (22%)	115 (30%)	
2 (FISH negative)	146 (27%)	40 (24%)	106 (28%)	
3	0 (0%)	0 (0%)	0 (0%)	
(Missing)	1	0	1	
Surgery				<0.001
Mastectomy	270 (50%)	60 (36%)	210 (55%)	
Lumpectomy	274 (50%)	105 (64%)	169 (45%)	
pCR	29 (19%)	29 (19%)	0	
Radiotherapy				0.046
No	29 (5.3%)	4 (2.4%)	25 (6.6%)	
Yes	514 (95%)	161 (98%)	353 (93%)	
(Missing)	1	0	1	
Endocrine therapy				0.2
No	24 (4.4%)	10 (6.1%)	14 (3.7%)	
Yes	519 (96%)	155 (94%)	364 (96%)	
(Missing)	1	0	1	

For continuous variables, values are expressed as median (minimum–maximum); for categorical variables, values are given as number (percentage). Tubule formation, mitotic count, and nuclear pleomorphism are the three individual components of the Elston–Ellis (Nottingham) grading system, each scored from 1 to 3; they are not the overall histological grade, which was 3 in all 544 patients as an inclusion criterion. *P* values are descriptive and characterize treatment selection in routine practice; balance for the weighted analysis is assessed by standardized mean differences (Figure 2).

BMI, body mass index; ER, estrogen receptor; HER2, human epidermal growth factor receptor 2; NST, no special type; pCR, pathologically documented complete response.

a Wilcoxon rank-sum test; Pearson’s chi-square test; Fisher’s exact one tailed test.

Candidate covariates were age, menopausal status, pathological subtype (no special type or others), ER and PR expression (<10%, 10%-80%, ≥80%), body mass index (BMI), clinical tumor size (T2 or T3) and nodal status (N0 or N+); missing data were handled by multiple imputation with chained equations. The final PS model retained age, menopausal status, pathological subtype, ER expression level, tumor size, and nodal status. Ki67 was not entered because it was missing for 135 of 544 patients (25%).

Inverse probability of treatment weighting (IPTW) was derived from the PS, as its inverse in the NAC group and the inverse of one minus the PS in the AC group, with truncation above the 99th percentile. Balance was assessed by standardized mean differences (SMDs) before and after weighting, with |SMD| < 0.10 indicating adequate balance (Figure 2).

**Figure 2:**
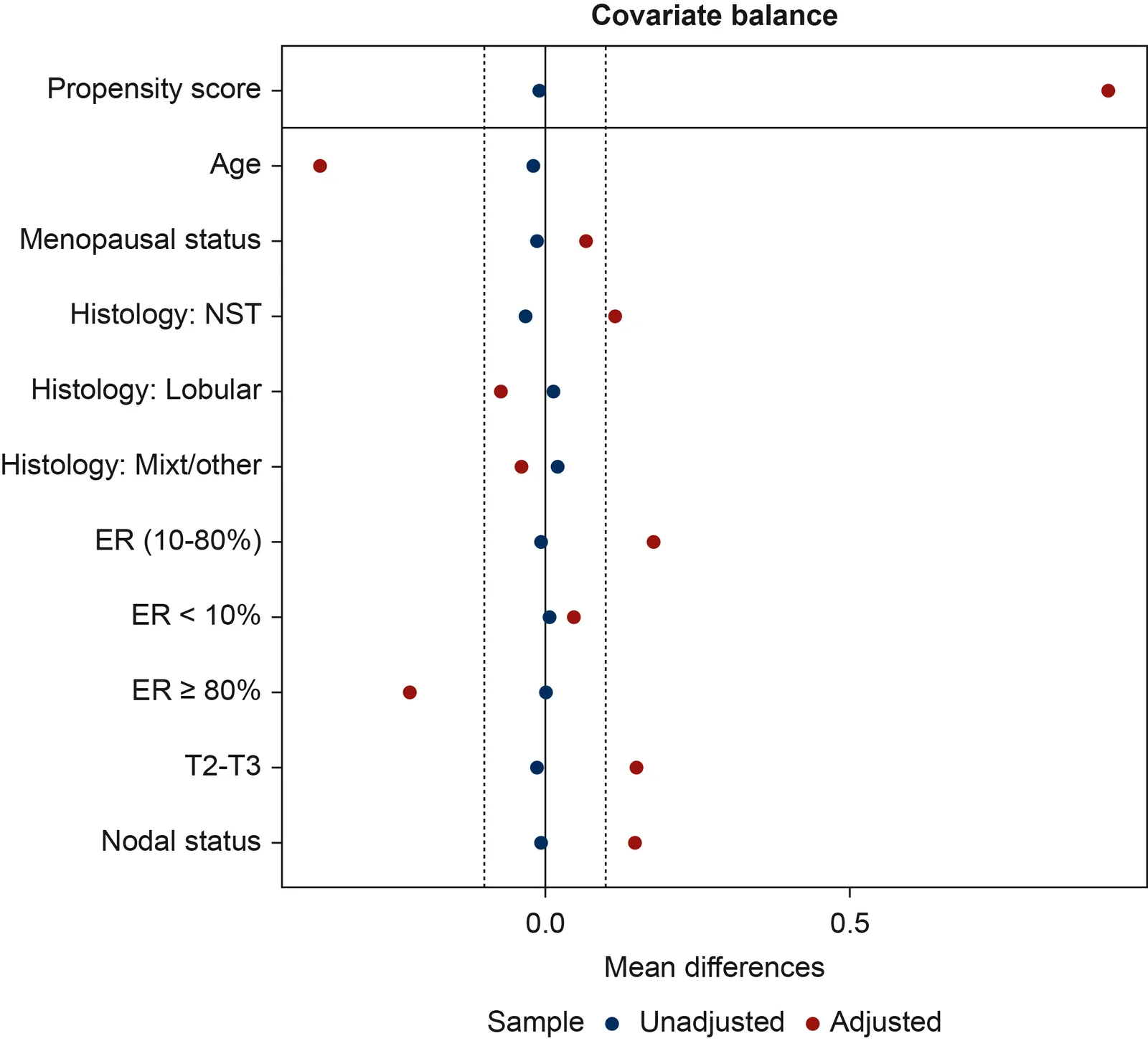
**Covariate balancing propensity score.** The SMD is a measure of distance between two group means for each variable. Because it is standardized, comparison across variables on different scales is realized in the same plot. Dashed lines indicate the conventional |SMD| = 0.10 threshold for adequate balance; all covariates were within this threshold after weighting. ER, estrogen receptor; NST, no special type; SMD, standardized mean difference; T2-T3, initial clinical tumor size group (>20 mm).

Survival was estimated by Kaplan–Meier curves and compared using log-rank tests in the unadjusted population and weighted univariate Cox models after IPTW.^26^ Multivariable Cox models included treatment strategy, menopausal status, clinical tumor size, nodal status, and type of surgery. Models before weighting were complete-case analyses including 486 patients; models after weighting used the imputed weighted dataset and included all 544 patients.

Interactions between treatment strategy and age, menopausal status, clinical tumor size, clinical and definitive nodal status, HER2-low status and PR expression were tested for OS and DRFS with adjustment for type of surgery, with a *P* value ≤0.10 being considered significant given limited power.^27^ For BCSS, LRFS, and BRFS, death without the event of interest is a competing risk; as our objective was etiological, cause-specific hazard models were fitted, and estimates should be interpreted as relative rates rather than absolute risks. The proportional hazards assumption was examined graphically and was not formally tested. Six correlated endpoints and 14 interaction terms were analyzed without adjustment for multiplicity. Tests were two-sided; analyses used R version 4.0.1 (Posit Software, PBC, Boston, Massachusetts).^28^

## Results

### Baseline descriptive analysis

The cohort comprised 544 patients with luminal B-like BC, of whom 165 received NAC and 379 AC (Figure 1). Baseline characteristics are detailed in Table 1. Patients in the NAC group were younger (median 51 compared with 54 years, *P* = 0.001), with no difference in menopausal status (*P* = 0.2) or BMI (*P* = 0.6). They more often had large tumors (T3: 28% compared with 13%, *P* < 0.001) and nodal involvement (N+: 39% compared with 22%, *P* < 0.001).

**Figure 1:**
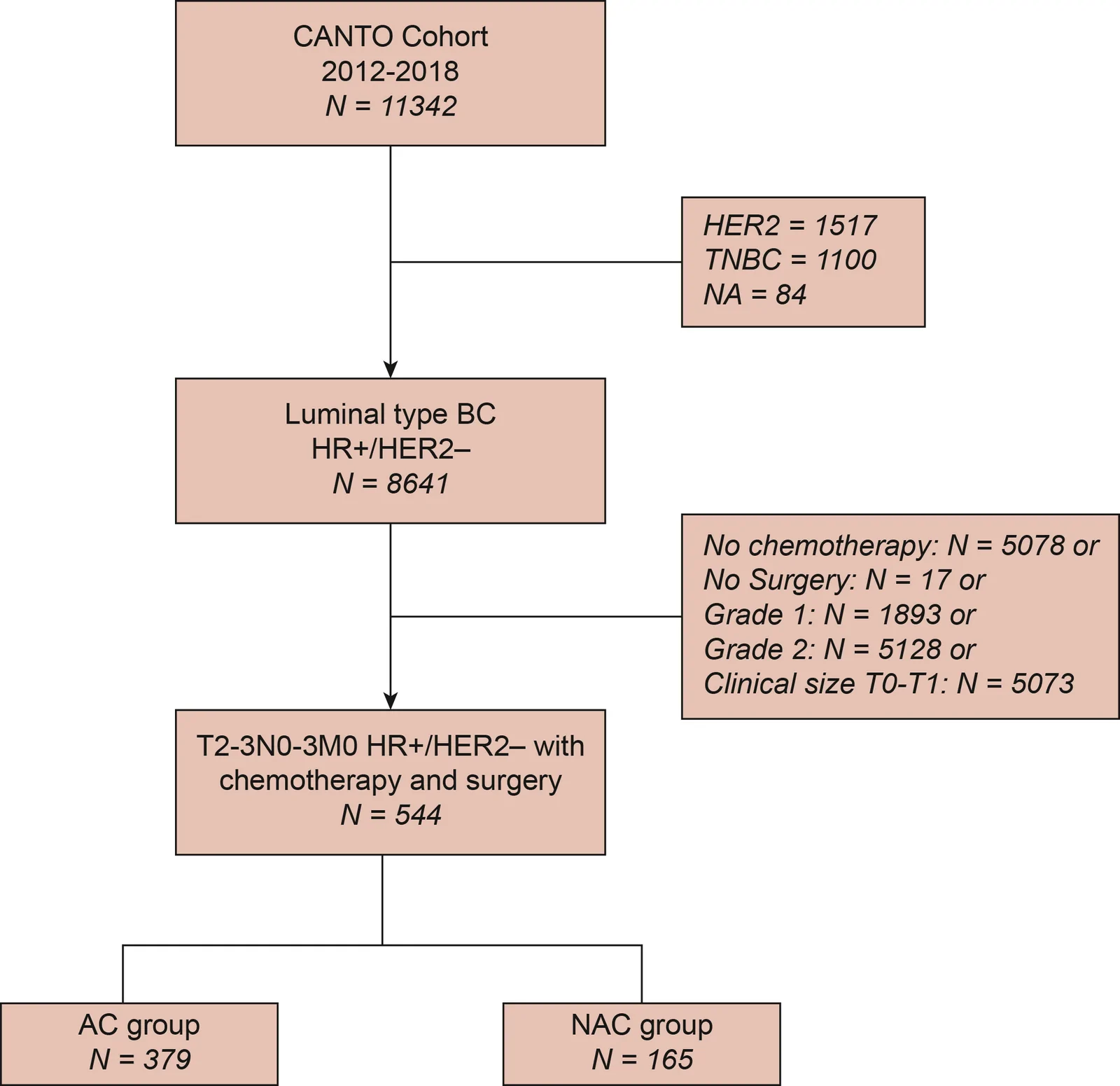
**Flowchart**. AC, adjuvant chemotherapy; BC, breast cancer; HER2, human epidermal growth factor receptor 2; HR, hormone receptor; NAC, neoadjuvant chemotherapy; TNBC, triple-negative breast cancer.

When available (*n* = 409), Ki67 was higher in the NAC group (median 42% compared with 34%, *P* = 0.002). Tumors with no special type were more frequent (90% compared with 78%, *P* = 0.002), with fewer lobular carcinomas, and the NAC group had lower ER (*P* < 0.001) and PR expression (*P* = 0.019) and a different distribution of HER2 level (HER2-0 in 54% compared with 42%, *P* = 0.023). Patients receiving NAC were therefore younger with poorer prognostic features, reflecting the selection that operates in routine practice.

Breast-conserving surgery was more frequent after NAC (64% compared with 45%, *P* < 0.001) and the pCR rate was 19% (29 of 165). Endocrine therapy was similar in both groups and radiotherapy was more frequent after NAC (98% compared with 93%, *P* = 0.046), consistent with the higher rate of breast conservation.

Supplementary analyses showed no significant difference between groups in hormonal risk factors (Supplementary Table S2, available at https://doi.org/10.1016/j.esmorw.2026.100789), comorbidities (Supplementary Table S3, available at https://doi.org/10.1016/j.esmorw.2026.100789), surgical methods, or endocrine therapy (Supplementary Tables S4-S6, available at https://doi.org/10.1016/j.esmorw.2026.100789). Chemotherapy was a sequential anthracycline–taxane regimen in 93% of patients, without difference in the number of cycles or dose reductions (Supplementary Table S5, available at https://doi.org/10.1016/j.esmorw.2026.100789).

### Survival analysis—before IPTW adjustment

Median follow-up was similar between the two groups, with a median of 72 months [interquartile range (IQR) 52, 85] in the NAC group and 73 months (IQR 52, 88) in the AC group (*P* = 0.9) (Supplementary Table S1, available at https://doi.org/10.1016/j.esmorw.2026.100789). Kaplan–Meier curves and multivariable Cox models before IPTW adjustment are presented in the supplementary data (Supplementary Figure S1 and Table S7, available at https://doi.org/10.1016/j.esmorw.2026.100789). Models before weighting were complete-case analyses including 486 of the 544 patients; 55 were excluded for missing nodal status and 3 for missing menopausal status. In the unadjusted analysis, OS at 60 months was 90% in the NAC group and 94% in the AC group (*P* = 0.0091; Supplementary Figure S1A, available at https://doi.org/10.1016/j.esmorw.2026.100789). BCSS at 60 months was 91% and 95%, respectively (*P* = 0.003; Supplementary Figure S1B, available at https://doi.org/10.1016/j.esmorw.2026.100789), IDFS 76% and 85% (*P* = 0.021; Supplementary Figure S1C, available at https://doi.org/10.1016/j.esmorw.2026.100789), and LRFS 95% and 99% (*P* = 0.0056; Supplementary Figure S1E, available at https://doi.org/10.1016/j.esmorw.2026.100789). DRFS at 60 months was 81% in the NAC group and 87% in the AC group (*P* = 0.077; Supplementary Figure S1D, available at https://doi.org/10.1016/j.esmorw.2026.100789).

In the multivariable Cox model before IPTW adjustment (*n* = 486), the hazard ratio for OS associated with NAC was 2.05 (95% CI 1.10-3.82, *P* = 0.024; Supplementary Table S7, available at https://doi.org/10.1016/j.esmorw.2026.100789). Menopausal status, tumor size, nodal involvement, and type of surgery were not significantly associated with OS. The hazard ratio was 1.72 (95% CI 1.12-2.65, *P* = 0.013) for IDFS (106 events), 4.23 (95% CI 1.57-11.4, *P* = 0.004) for LRFS (19 events), and 1.41 (95% CI 0.88-2.26, *P* = 0.2) for DRFS (90 events).

### Survival analysis—propensity score and IPTW adjustment

The PS was estimated from age, menopausal status, histological type, ER expression level, clinical tumor size and nodal status; its distribution is shown in Supplementary Figure S2 (available at https://doi.org/10.1016/j.esmorw.2026.100789) and standardized SMDs in Figure 2, all covariates being within the 0.10 threshold after weighting.

After IPTW adjustment, the hazard ratio for OS associated with NAC was 1.36 (95% CI 0.94-1.97, *P* = 0.10; 54 events; Figure 3A). The hazard ratio was 1.51 (95% CI 1.01-2.27, *P* = 0.047; 46 events) for BCSS (Figure 3B), 1.21 (95% CI 0.94-1.55, *P* = 0.14; 118 events) for IDFS (Figure 3C), and 1.05 (95% CI 0.80-1.38, *P* = 0.70; 100 events) for DRFS (Figure 3D). The hazard ratio for LRFS was 2.29 (95% CI 1.15-4.58, *P* = 0.018; Figure 3E) and that for BRFS 7.68 (95% CI 1.09-54.2, *P* = 0.041; Figure 3F). These two estimates were based on 19 and 6 events, respectively, and are therefore very imprecise; the width of the BRFS interval in particular precludes any estimate of the magnitude of the association and this endpoint should be regarded as hypothesis-generating only.

**Figure 3:**
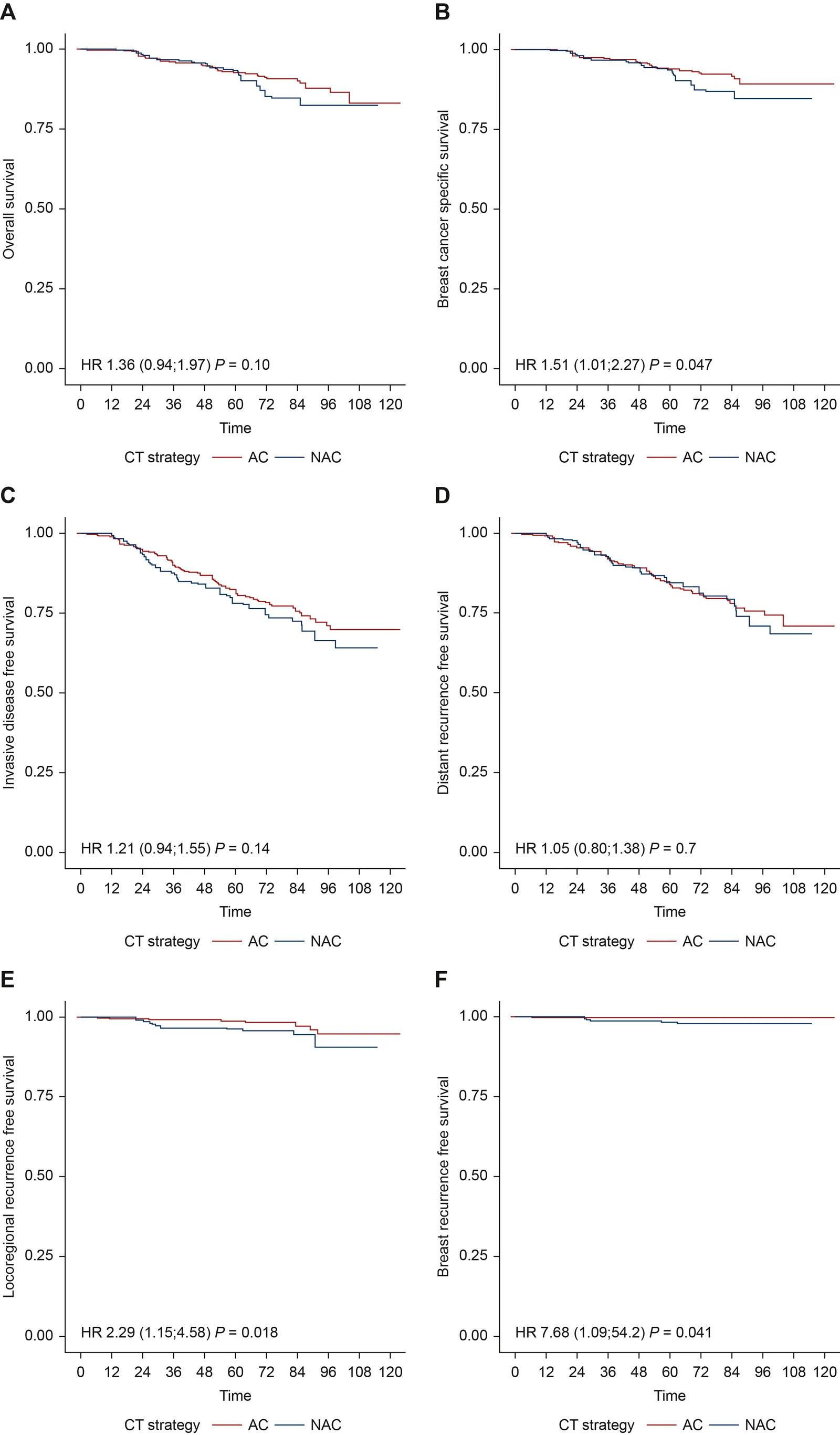
**Survival according to the chemotherapy strategy after IPTW adjustment.** (A) Overall survival. (B) Breast cancer-specific survival. (C) Invasive disease-free survival. (D) Distant recurrence-free survival. (E) Locoregional recurrence-free survival. (F) Breast recurrence-free survival. HRs (with 95% confidence intervals shown in square brackets) and *P* values were calculated from Cox univariate models after IPTW adjustment. AC, adjuvant chemotherapy; CT, chemotherapy; HR, hazard ratio; IPTW, inverse probability of treatment weighting; NAC, neoadjuvant chemotherapy.

In the IPTW-adjusted multivariable model, the hazard ratio for OS associated with NAC was 1.62 (95% CI 1.10-2.38, *P* = 0.014); the corresponding weighted univariable estimate was 1.36 (95% CI 0.94-1.97, *P* = 0.10). The hazard ratio for IDFS was 1.43 (95% CI 1.10-1.85, *P* = 0.007). The hazard ratio was 1.31 (95% CI 0.99-1.74, *P* = 0.062) for DRFS and 2.84 (95% CI 1.39-5.80, *P* = 0.004) for LRFS. These models include type of surgery, which is a variable determined after the treatment decision, and the difference between the weighted univariable and multivariable estimates should be read with this in mind.

Lumpectomy was associated with lower hazard ratios than mastectomy for OS (0.53, 95% CI 0.36-0.78, *P* = 0.001), DRFS (0.42, 95% CI 0.31-0.56, *P* < 0.001), IDFS (0.51, 95% CI 0.40-0.67, *P* < 0.001), and LRFS (0.47, 95% CI 0.24-0.91, *P* = 0.026) (Table 2). This association is confounded by indication and should not be interpreted causally. Interaction tests, adjusted for type of surgery, were carried out for OS and DRFS. For OS, interactions reached the prespecified threshold for age (*P* < 0.01), menopausal status (*P* = 0.05), clinical tumor size (*P* = 0.08), clinical nodal status (*P* < 0.01), definitive nodal status (*P* < 0.01) and PR expression (*P* < 0.01), but not HER2-low status (*P* = 0.52). The hazard ratio for NAC was 2.39 (95% CI 1.44-3.97) in cN0 compared with 0.83 (95% CI 0.46-1.51) in cN+ tumors and 1.94 (95% CI 1.24-3.04) in cT2 compared with 0.88 (95% CI 0.42-1.87) in cT3 tumors. For DRFS, interactions were significant for menopausal status (*P* = 0.02), tumor size (*P* < 0.01), and nodal status (*P* = 0.05 and *P* < 0.01), in the same direction. The interaction with definitive nodal status should not be read causally, as this variable is modified by NAC. These analyses are exploratory.

**Table 2:** Cox multivariate models after IPTW adjustment

Variable	HR	95% CI	*P* value
OS (*n* = 544, 54 events)			
Chemotherapy			
AC	1.00	—	
NAC	1.62	1.10-2.38	0.014
Surgery			
Mastectomy	1.00	—	
Lumpectomy	0.53	0.36-0.78	0.001
DRFS (*n* = 544, 100 events)			
Chemotherapy			
AC	1.00	—	
NAC	1.31	0.99-1.74	0.062
Surgery			
Mastectomy	1.00	—	
Lumpectomy	0.42	0.31-0.56	<0.001
IDFS (*n* = 544, 118 events)			
Chemotherapy			
AC	1.00	—	
NAC	1.43	1.10-1.85	0.007
Surgery			
Mastectomy	1.00	—	
Lumpectomy	0.51	0.40-0.67	<0.001
LRFS (*n* = 544, 19 events)			
Chemotherapy			
AC	1.00	—	
NAC	2.84	1.39-5.80	0.004
Surgery			
Mastectomy	1.00	—	
Lumpectomy	0.47	0.24-0.91	0.026

AC, adjuvant chemotherapy; CI, confidence interval; DRFS, distant recurrence-free survival; HR, hazard ratio; IDFS, invasive disease-free survival; IPTW, inverse probability of treatment weighting; LRFS, locoregional recurrence-free survival; NAC, neoadjuvant chemotherapy; OS, overall survival.

## Discussion

In this prospective multicentric cohort of grade 3 HR-positive/HER2-negative T2–3N0–3M0 tumors, NAC was associated with a higher rate of locoregional recurrence than AC, while no difference was observed in DRFS; differences in OS and IDFS appeared in some but not all model specifications. We do not interpret the locoregional finding as the explanation for the mortality estimates: isolated locoregional recurrence accounts for a small fraction of BC deaths and the endpoint most closely linked to mortality showed no difference.^29^^,^^30^ The two observations are more plausibly independent, with the locoregional finding reflecting the surgical consequences of the neoadjuvant sequence and the mortality estimates remaining vulnerable to residual confounding by indication.

The interaction analyses are the most clinically interpretable element of this study. The excess risk associated with NAC was concentrated in cN0 and cT2 disease in patients aged ≥40 years and with preserved PR expression, that is, in those for whom a neoadjuvant indication is least compelling; no disadvantage was apparent in cT3 or node-positive disease or in patients younger than 40, where NAC is usually proposed and point estimates were close to or below unity. This is what would be expected if the association reflected, at least in part, the use of NAC outside its established indications rather than a deleterious effect of the sequence itself and it is equally compatible with residual confounding operating differentially across subgroups.

Our results should be read alongside available evidence. The Early Breast Cancer Trialists’ Collaborative Group meta-analysis^29^ of 10 randomized trials found no difference between NAC and AC in distant recurrence, BC mortality, or overall mortality, but a 15-year absolute excess of local recurrence of 5.5% (95% CI 2.4-8.6; 21.4% compared with 15.9%) in the neoadjuvant arms, attributed largely to greater use of breast-conserving surgery; the National Surgical Adjuvant Breast and Bowel Project protocols B-18 and B-27 likewise reported no survival difference.^2^^,^^9^^,^^31^^,^^32^ Among registry analyses restricted to HR-positive/HER2-negative disease, a Swedish matched study found no difference,^20^ whereas a SEER analysis of T2N1M0 tumors reported inferior survival with NAC, concentrated in patients without pCR.^21^ Our findings fall between these and the heterogeneity suggests that the treatment effect, if any, is small relative to the confounding separating the two populations in practice.

Chemoresistance is a central concern in luminal B-like tumors, as reflected by their modest pCR rates. Cortazar et al.^1^ showed that pCR was not related to survival in ER-positive/HER2-negative grade 1/2 tumors, unlike grade 3 and ER-negative tumors, suggesting that the ER pathway is a cornerstone of chemoresistance. Hamy et al.^33^ found ER pathway gene expression inversely correlated with pCR. A pCR rate of 19% means that four in five patients treated with NAC underwent surgery with residual invasive disease, obtaining neither the prognostic information nor the escalation opportunity that justify the neoadjuvant sequence in TNBC and HER2-positive disease. We did not compare outcomes by pathological response within the NAC group because it is a posttreatment variable with no counterpart in the AC group.

Strategies to increase pathological response rates in this subtype, including the addition of immune checkpoint inhibitors or stereotactic radiotherapy with adenosine pathway blockade, are under investigation but do not address the question of treatment sequencing considered here.^34^, ^35^, ^36^ No validated tool currently identifies, before treatment, which grade 3 luminal B-like tumors will respond to chemotherapy. Multigene assays inform the decision to give chemotherapy at all, intrinsic subtyping has been applied in the neoadjuvant setting, and multiomic and machine-learning classifiers have been proposed for pCR prediction but none has been prospectively validated for the sequencing decision itself.

Treatment delay has been proposed to account for poorer outcomes after NAC in chemoresistant tumors, as time to surgery is associated with survival in registry analyses.^37^^,^^38^ We did not analyze treatment intervals and can offer no evidence for or against this mechanism.

The strengths of this analysis are the prospective multicentric design of CANTO, with centrally collected data from 26 centers, a 6-year median follow-up, and the joint analysis of locoregional and distant endpoints. IPTW is an established method for estimating average treatment effects from observational data^39^^,^^40^ and has been used in BC studies.^41^^,^^42^

Endocrine therapy alone has shown efficacy in luminal B-like disease when guided by genomic and biological response, as in ADAPT, where patients with a low recurrence score and a favorable response to short-term endocrine therapy had excellent outcomes without chemotherapy.^43^^,^^44^ Alternative endpoints such as the Clinical-Pathologic Stage + Estrogen receptor status and Grade score may also be useful when evaluating NAC in luminal BC.^15^^,^^45^

Several limitations apply. This is an observational comparison of two strategies that were not randomly allocated; weighting on six pretreatment covariates reduces but cannot eliminate confounding by indication, in a direction that disadvantages the NAC group. Ki67 index and HER2 expression level were not included in the PS: Ki67 data were missing for 135 of 544 patients (25%) and imputing a continuous prognostic variable at this level of missingness within a treatment-selection model was judged too risky; where measured, Ki67 was higher in the NAC group (median 42% compared with 34%). As Ki67 is associated with chemosensitivity, its omission leaves a known confounder unadjusted and part of the association reported may reflect this imbalance. Defining luminal B-like status by grade alone, without Ki67 or genomic data for the entire cohort, may have introduced misclassification. The number of locoregional (19) and breast recurrence (6) events were small and the corresponding estimates were fragile; 6 correlated endpoints and 14 interaction terms were tested without adjustment for multiplicity, and the proportional hazards assumption was not formally tested. Finally, weighted Cox models estimate an average, prognostic association, whereas the clinical question is predictive: which patients derive differential benefit from one sequence rather than the other. Our interaction analyses are a first step but remain exploratory and underpowered.

Taken together, our data do not support the routine use of NAC in cN0, cT2 luminal B-like BC in the absence of an anticipated surgical benefit. The increased risk of local recurrence suggests careful monitoring and possibly adjusted surgical strategies when NAC is used.

These findings advocate a tailored approach, taking into account tumor biology and patient-specific factors. They add one element to a body of evidence that remains heterogeneous and cannot by themselves determine the sequencing of chemotherapy in this population.

## Supplementary data

Supplementary Figure S1 AB

Supplementary Figure S1 CD

Supplementary Figure S1 EF
