# Supplementary material for: Neoadjuvant versus adjuvant chemotherapy in luminal B-like breast cancer: a subset analysis of the CANTO study
Source: ESMO Real World Data Digit Oncol. 2026 Oct 12;14:100789. doi: 10.1016/j.esmorw.2026.100789 (PMC13682014; doi:10.1016/j.esmorw.2026.100789)
Supplement: Supplementary file 1 [file mmc1.pdf]

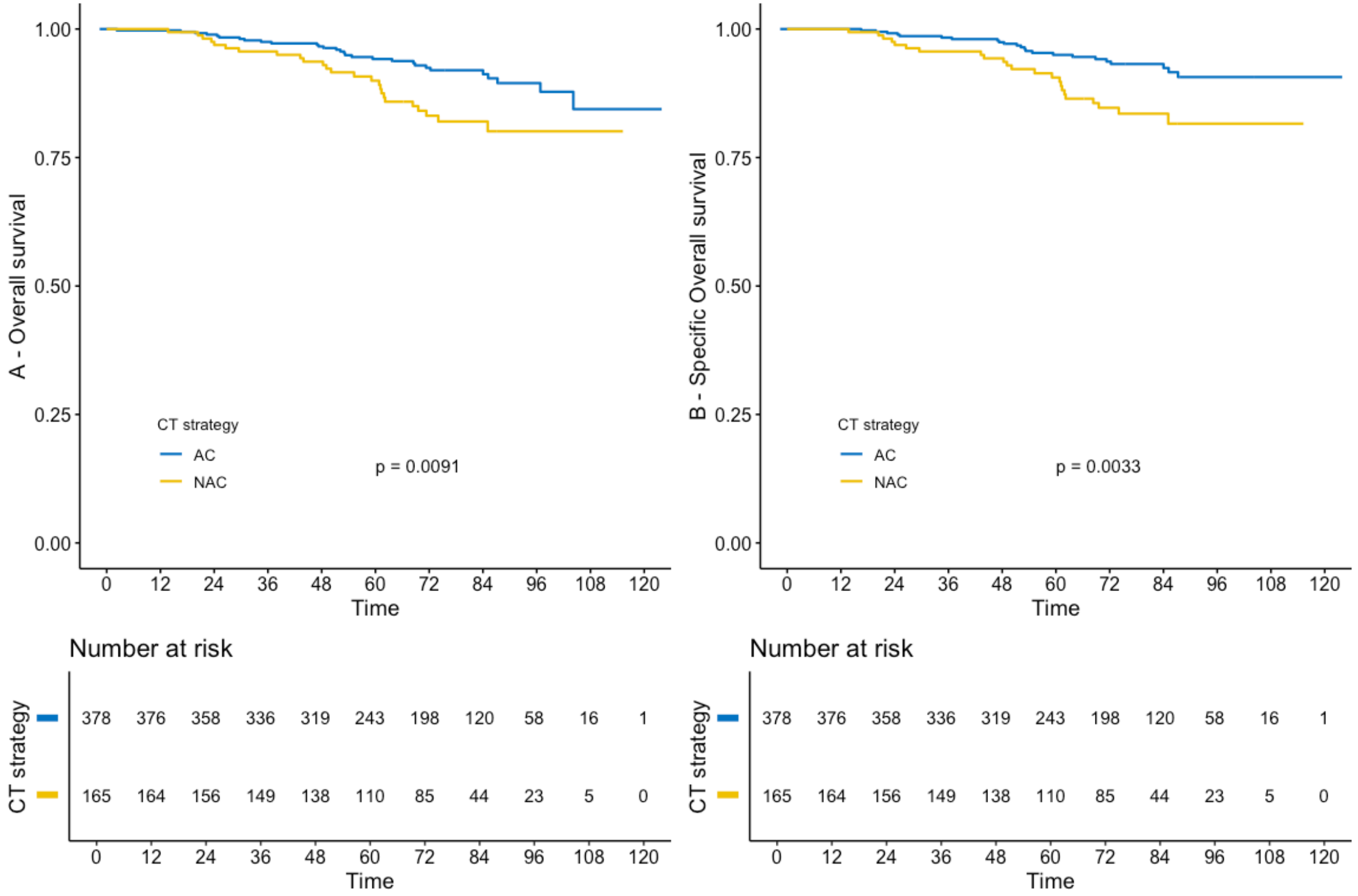

A - Overall survival
1.00
0.75
0.50
0.25
0.00
CT strategy
AC
NAC
p = 0.0091
0
12
24
36
48
60
72
84
96
108
120
Time
Number at risk
CT strategy
378 376 358 336 319 243 198 120 58 16 1
165 164 156 149 138 110 85 44 23 5 0
B - Specific Overall survival
p = 0.0033
