# Supplementary material for: Neoadjuvant versus adjuvant chemotherapy in luminal B-like breast cancer: a subset analysis of the CANTO study
Source: ESMO Real World Data Digit Oncol. 2026 Oct 12;14:100789. doi: 10.1016/j.esmorw.2026.100789 (PMC13682014; doi:10.1016/j.esmorw.2026.100789)
Supplement: Supplementary file 2 [file mmc2.pdf]

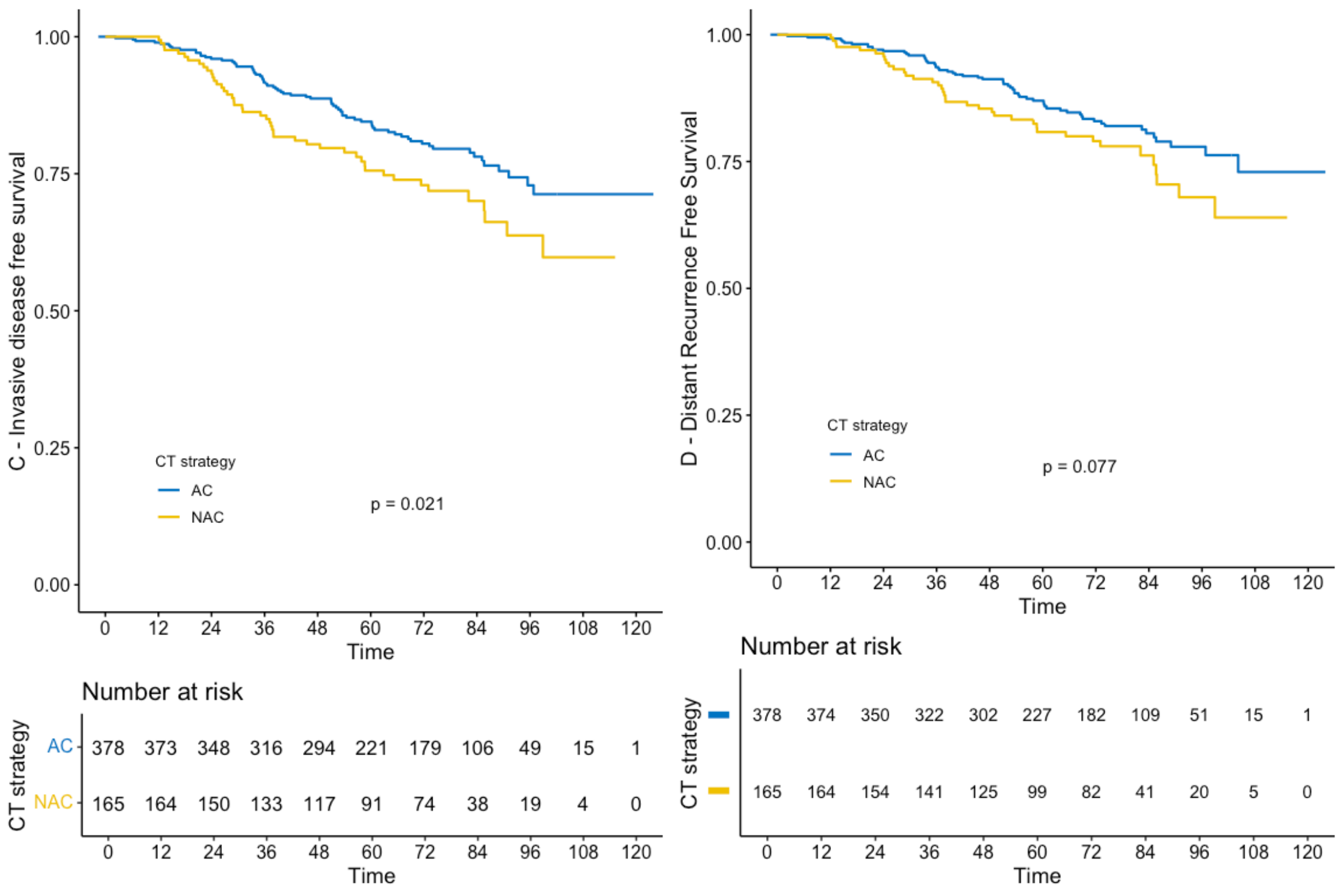

C - Invasive disease free survival
1.00
0.75
0.50
0.25
0.00
CT strategy
AC
NAC
p = 0.021
0 12 24 36 48 60 72 84 96 108 120
Time
Number at risk
CT strategy
AC 378 373 348 316 294 221 179 106 49 15 1
NAC 165 164 150 133 117 91 74 38 19 4 0
0 12 24 36 48 60 72 84 96 108 120
Time
D - Distant Recurrence Free Survival
1.00
0.75
0.50
0.25
0.00
CT strategy
AC
NAC
p = 0.077
0 12 24 36 48 60 72 84 96 108 120
Time
Number at risk
CT strategy
378 374 350 322 302 227 182 109 51 15 1
165 164 154 141 125 99 82 41 20 5 0
0 12 24 36 48 60 72 84 96 108 120
Time
