# Supplementary material for: Neoadjuvant versus adjuvant chemotherapy in luminal B-like breast cancer: a subset analysis of the CANTO study
Source: ESMO Real World Data Digit Oncol. 2026 Oct 12;14:100789. doi: 10.1016/j.esmorw.2026.100789 (PMC13682014; doi:10.1016/j.esmorw.2026.100789)
Supplement: Supplementary file 3 [file mmc3.pdf]

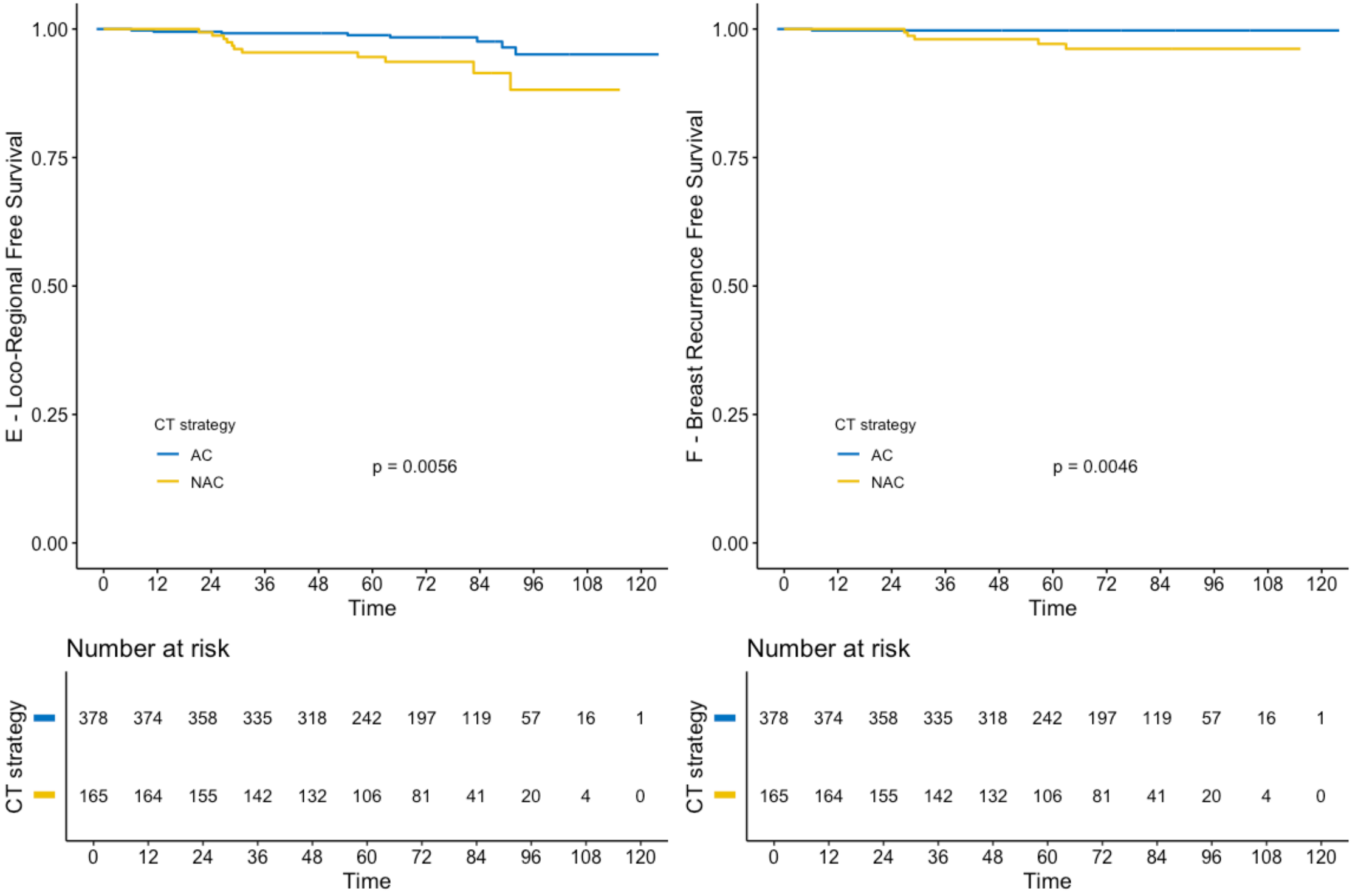
E - Loco-Regional Free Survival
1.00
0.75
0.50
0.25
0.00
CT strategy
AC
NAC
p = 0.0056
0 12 24 36 48 60 72 84 96 108 120
Time
Number at risk
CT strategy
378 374 358 335 318 242 197 119 57 16 1
165 164 155 142 132 106 81 41 20 4 0
0 12 24 36 48 60 72 84 96 108 120
Time
F - Breast Recurrence Free Survival
1.00
0.75
0.50
0.25
0.00
CT strategy
AC
NAC
p = 0.0046
0 12 24 36 48 60 72 84 96 108 120
Time
Number at risk
CT strategy
378 374 358 335 318 242 197 119 57 16 1
165 164 155 142 132 106 81 41 20 4 0
0 12 24 36 48 60 72 84 96 108 120
Time
