# Supplementary material for: Neoadjuvant versus adjuvant chemotherapy in luminal B-like breast cancer: a subset analysis of the CANTO study
Source: ESMO Real World Data Digit Oncol. 2026 Oct 12;14:100789. doi: 10.1016/j.esmorw.2026.100789 (PMC13682014; doi:10.1016/j.esmorw.2026.100789)
Supplement: Supplementary file 4 [file mmc4.pdf]

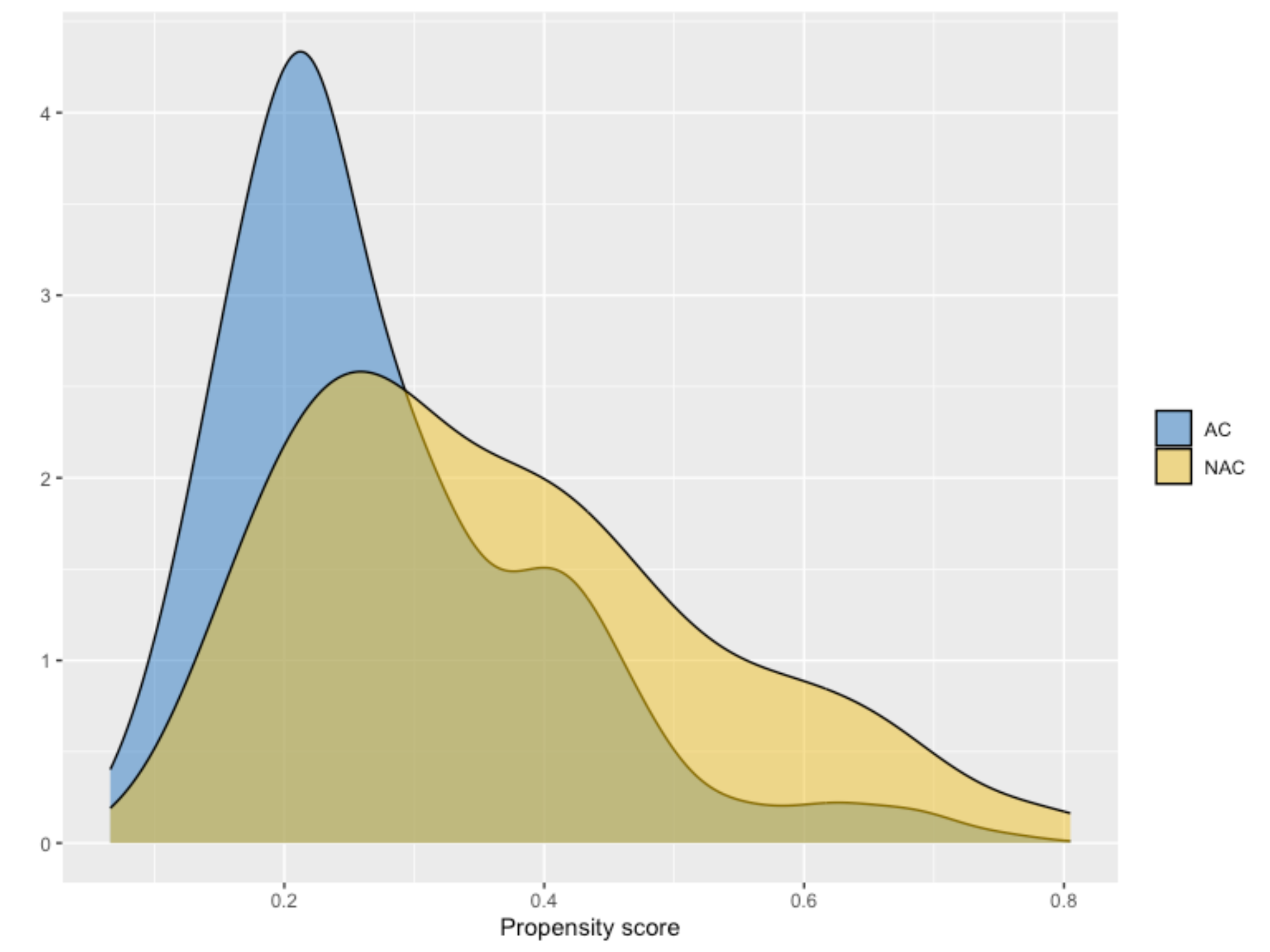
4
3
2
1
0
0.2
0.4
0.6
0.8
Propensity score
AC
NAC
