# Supplementary material for: Neoadjuvant versus adjuvant chemotherapy in luminal B-like breast cancer: a subset analysis of the CANTO study
Source: ESMO Real World Data Digit Oncol. 2026 Oct 12;14:100789. doi: 10.1016/j.esmorw.2026.100789 (PMC13682014; doi:10.1016/j.esmorw.2026.100789)
Supplement: Supplementary file 5 [file mmc5.docx]

**Supplementary**

Table S1 – Median follow-up

| **Characteristic** | **Median Follow up in months (IQR)** | **p-value** |
| --- | --- | --- |
| **Chemotherapy** |  | 0.9 |
| NAC | 72 (52, 85) |  |
| AC | 73 (52, 88) |  |

*NAC: Neoadjuvant Chemotherapy*

*AC: Adjuvant Chemotherapy*

Table S2 – Description of hormonal risk factors

| **Variable** | **Overall**, N = 544 | **NAC**, N = 165 | **AC**, N = 379 | **p-value** |
| --- | --- | --- | --- | --- |
| **Age of first period** | 13.00 (9.00, 18.00) | 13.00 (10.00, 17.00) | 13.00 (9.00, 18.00) | 0.9 |
| (Missing) | 110 | 31 | 79 |  |
| **Pregnancies (n)** | 2.00 (0.00, 11.00) | 2.00 (0.00, 7.00) | 2.00 (0.00, 11.00) | 0.2 |
| (Missing) | 1 | 0 | 1 |  |
| **Age of delivery status** | 26.0 (16.0, 45.0) | 27.0 (16.0, 45.0) | 26.0 (16.0, 44.0) | 0.4 |
| (Missing) | 210 | 65 | 145 |  |
| **Child (n)** | 13.00 (9.00, 18.00) | 2.00 (0.00, 6.00) | 2.00 (0.00, 7.00) | 0.014 |
| (Missing) | 110 | 43 | 107 |  |
|  |  |  |  |  |
| **Number of breast feeding** | 2.00 (1.00, 6.00) | 2.00 (1.00, 3.00) | 2.00 (1.00, 6.00) | 0.5 |
| (Missing) | 350 | 108 | 242 |  |
| **Breast feeding duration** | 3.0 (0.3, 38.0) | 3.0 (1.0, 38.0) | 3.0 (0.3, 36.0) | 0.031 |
| (Missing) | 357 | 111 | 246 |  |
| **Contraception** | 141 (53%) | 48 (56%) | 93 (52%) | 0.6 |
| (Missing) | 279 | 79 | 200 |  |
| **Age of menopauses** | 50.5 (30.0, 63.0) | 50.5 (39.0, 58.0) | 50.5 (30.0, 63.0) | 0.6 |
| (Missing) | 292 | 95 | 197 |  |
| **Hormone replacement therapy (HRT)** |  |  |  | >0.9 |
| No | 188 (74%) | 52 (74%) | 136 (74%) |  |
| Yes | 66 (26%) | 18 (26%) | 48 (26%) |  |
| (Missing) | 290 | 95 | 195 |  |
| **Duration of HRT** | 5.0 (0.0, 25.0) | 3.5 (0.0, 13.0) | 6.0 (0.0, 25.0) | 0.11 |
| (Missing) | 495 | 149 | 346 |  |
| *For continuous variables, values are expressed as median (minimum–maximum); for categorical variables, values are given as number (percentage).* | | | | |

Table S3 – Description of comorbidities

| **Variable** | **Overall**, N = 544 | **NAC**, N = 165 | **AC**, N = 379 | **p-value** |  |
| --- | --- | --- | --- | --- | --- |
| **ECOG** |  |  |  | 0.081 |  |
| 0 | 489 (97%) | 144 (95%) | 345 (98%) |  |  |
| 1 | 15 (3.0%) | 8 (5.3%) | 7 (2.0%) |  |  |
| 2 | 0 (0%) | 0 (0%) | 0 (0%) |  |  |
| 3 | 0 (0%) | 0 (0%) | 0 (0%) |  |  |
| 4 | 0 (0%) | 0 (0%) | 0 (0%) |  |  |
| (Missing) | 40 | 13 | 27 |  |  |
| **Charlson Comorbidity Index** |  |  |  | 0.3 |  |
| 0 | 394 (84%) | 128 (88%) | 266 (82%) |  |  |
| 1 | 48 (10%) | 11 (7.6%) | 37 (11%) |  |  |
| 2 | 18 (3.8%) | 5 (3.4%) | 13 (4.0%) |  |  |
| ≥3 | 9 (1.9%) | 1 (0.7%) | 8 (2.5%) |  |  |
| (Missing) | 75 | 20 | 55 |  |  |
| **Smoking status** |  |  |  | 0.5 |  |
| Active | 97 (18%) | 32 (20%) | 65 (17%) |  |  |
| Former | 121 (22%) | 40 (25%) | 81 (21%) |  |  |
| Never | 323 (60%) | 91 (56%) | 232 (61%) |  |  |
| (Missing) | 3 | 2 | 1 |  |  |
| **Smoking pack years** | 19 (0, 96) | 18 (0, 96) | 13 (1, 91) | 0.2 |  |
| (Missing) | 391 | 115 | 276 |  |  |
| **Daily alcohol consumption** |  |  |  | 0.2 |  |
| No | 473 (90%) | 147 (92%) | 326 (89%) |  |  |
| Yes | 54 (10%) | 12 (7.5%) | 42 (11%) |  |  |
| (Missing) | 17 | 6 | 11 |  |  |
| **Osteoporosis** |  |  |  | 0.2 |  |
| No | 429 (93%) | 132 (96%) | 297 (92%) |  |  |
| Yes | 31 (6.7%) | 6 (4.3%) | 25 (7.8%) |  |  |
| (Missing) | 84 | 27 | 57 |  |  |
| **Cardiac function:**  **Left Ventricular Ejection Fraction** | 65 (47, 84) | 66 (50, 82) | 65 (47, 84) | 0.6 |  |
| (Missing) | 152 | 32 | 120 |  |  |
| *For continuous variables, values are expressed as median (minimum–maximum); for categorical variables, values are given as number (percentage).* | | | | | |

Table S4 – Description of surgical procedures

| **Variable** | **Overall**, N = 544 | **NAC**, N = 165 | **AC**, N = 379 | **p-value** |
| --- | --- | --- | --- | --- |
| **Subcutaneous mastectomy** | 18 (3.3%) | 6 (3.6%) | 12 (3.2%) | 0.8 |
| **Immediate reconstruction** | 19 (3.5%) | 8 (4.8%) | 11 (2.9%) | 0.3 |
| **Sentinel Lymph Node** | 256 (47%) | 66 (40%) | 190 (50%) | 0.030 |
| **Axillary dissection** | 374 (69%) | 122 (74%) | 252 (66%) | 0.085 |
| **Surgical revision** | 43 (7.9%) | 11 (6.7%) | 32 (8.4%) | 0.5 |
| **Complication** | 107 (20%) | 29 (18%) | 78 (21%) | 0.4 |
| (Missing) | 1 | 0 | 1 |  |

Table S5 – Description of chemotherapy

| **Variable** | **Overall**, N = 544 | **NAC**, N = 165 | **AC**, N = 379 |
| --- | --- | --- | --- |
| **Clinical trial** | 149 (27%) | 49 (30%) | 100 (26%) |
| **Number of chemotherapy cycles** | 6.00 (1.00, 16.00) | 7.00 (4.00, 16.00) | 6.00 (1.00, 15.00) |
| (Missing) | 1 | 0 | 1 |
| **Chemotherapy Sequence** |  |  |  |
| Single regimen | 40 (7.4%) | 8 (4.8%) | 32 (8.4%) |
| Sequential two-regimen | 504 (93%) | 157 (95%) | 347 (92%) |
| **AC group - First sequence of chemotherapy** |  |  |  |
| FEC | 302 (80%) | 0 (NA%) | 302 (80%) |
| FAC | 5 (1.3%) | 0 (NA%) | 5 (1.3%) |
| ET | 2 (0.5%) | 0 (NA%) | 2 (0.5%) |
| A | 2 (0.5%) | 0 (NA%) | 2 (0.5%) |
| C | 2 (0.5%) | 0 (NA%) | 2 (0.5%) |
| Cape | 56 (15%) | 0 (NA%) | 56 (15%) |
| Cpt | 10 (2.6%) | 0 (NA%) | 10 (2.6%) |
| (Missing) | 165 | 165 | 0 |
| **AC group - Second sequence of chemotherapy** |  |  |  |
| ET | 1 (0.3%) | 0 (NA%) | 1 (0.3%) |
| T | 344 (99%) | 0 (NA%) | 344 (99%) |
| A | 2 (0.6%) | 0 (NA%) | 2 (0.6%) |
| (Missing) | 197 | 165 | 32 |
| **NAC group – First sequence of chemotherapy** |  |  |  |
| FEC | 115 (70%) | 115 (70%) | 0 (NA%) |
| FAC | 21 (13%) | 21 (13%) | 0 (NA%) |
| AC | 4 (2.4%) | 4 (2.4%) | 0 (NA%) |
| ET | 1 (0.6%) | 1 (0.6%) | 0 (NA%) |
| AI | 24 (15%) | 24 (15%) | 0 (NA%) |
| (Missing) | 379 | 0 | 379 |
| **NAC group – Second sequence of chemotherapy** |  |  |  |
| FAC | 1 (0.6%) | 1 (0.6%) | 0 (NA%) |
| ET | 155 (99%) | 155 (99%) | 0 (NA%) |
| Cape | 1 (0.6%) | 1 (0.6%) | 0 (NA%) |
| (Missing) | 387 | 8 | 379 |
| **Reduction of dose of at least a cycle** |  |  |  |
| No | 475 (88%) | 145 (88%) | 330 (88%) |
| Yes | 67 (12%) | 20 (12%) | 47 (12%) |
| (Missing) | 2 | 0 | 2 |
| **Number or cycle with dose reduction** |  |  |  |
| 1 | 34 (51%) | 14 (70%) | 20 (43%) |
| 2 | 21 (31%) | 5 (25%) | 16 (34%) |
| 3 | 8 (12%) | 1 (5.0%) | 7 (15%) |
| 4 | 1 (1.5%) | 0 (0%) | 1 (2.1%) |
| 5 | 3 (4.5%) | 0 (0%) | 3 (6.4%) |
| (Missing) | 477 | 145 | 332 |
| **Targeted therapy** | 8 (1.5%) | 4 (2.4%) | 4 (1.1%) |
| **Targeted therapies** |  |  |  |
| Bevacizumab | 1 (12%) | 1 (25%) | 0 (0%) |
| Denosumab | 2 (25%) | 0 (0%) | 2 (50%) |
| Everolimus | 2 (25%) | 1 (25%) | 1 (25%) |
| Olaparib/placebo | 1 (12%) | 0 (0%) | 1 (25%) |
| Palbociclib | 2 (25%) | 2 (50%) | 0 (0%) |
| (Missing) | 536 | 161 | 375 |

*For continuous variables, values are expressed as median (minimum–maximum); for categorical variables, values are given as number (percentage).*

Table S6 – Description of adjuvant endocrine therapy

| **Variable** | **Overall**, N = 544 | **NAC**, N = 165 | **AC**, N = 379 | **p-value** |
| --- | --- | --- | --- | --- |
| **Type of endocrine therapy** |  |  |  | 0.064 |
| Anti-estrogen (Tamoxifen) (+/- LH-RH) | 162 (30%) | 60 (36%) | 102 (27%) |  |
| Aromatase inhibitors (+/- LH-RH) | 260 (48%) | 71 (43%) | 189 (50%) |  |
| Aromatase inhibitors + Tamoxifen (+/- LH-RH) | 91 (17%) | 22 (13%) | 69 (18%) |  |
| LH-RH agonist alone | 4 (0.7%) | 2 (1.2%) | 2 (0.5%) |  |
| No hormone therapy | 24 (4.4%) | 10 (6.1%) | 14 (3.7%) |  |
| (Missing) | 3 | 0 | 3 |  |
| **On-going endocrine therapy at time of data lock** |  |  |  | 0.041 |
| No | 218 (42%) | 76 (49%) | 142 (39%) |  |
| Yes | 298 (58%) | 79 (51%) | 219 (61%) |  |
| (Missing) | 28 | 10 | 18 |  |
| **Total duration of hormonotherapy (months)** | 56 (1, 106) | 53 (1, 105) | 59 (1, 106) | 0.037 |
| (Missing) | 28 | 10 | 18 |  |

*FEC (Fluorouracil, Epirubicin, and Cyclophosphamide)*

*FAC (Fluorouracil, Doxorubicin (Adriamycin), and Cyclophosphamide)*

*AC (Doxorubicin (Adriamycin) and Cyclophosphamide)*

*A (Adriamycin)*

*A-T (Adriamycin (Doxorubicin) and Taxotere (Docetaxel))*

*C (Cyclophosphamide)*

*T (Taxane: docetaxel or paclitaxel); Cape (Capecitabine)*

*CPT-T (Irinotecan (CPT-11) and Taxotere (Docetaxel))*

*For continuous variables, values are expressed as median (minimum–maximum); for categorical variables, values are given as number (percentage).*

Figure S1 - Survival according to the chemotherapy strategy before inverse probability of treatment weighting adjustment.

A. Overall survival
B. Specific overall survival
C. Invasive disease free survival
D. Distant recurrence free survival
E. Loco-regional free survival
F. Breast Recurrence free survival

Table S7 – Cox multivariable analyses before inverse probability of treatment weighting adjustment.

| **Overall Survival** | **N** | **Event N** | **HR** | **95% CI** | **p-value** |
| --- | --- | --- | --- | --- | --- |
| **Chemotherapy** | 486 | 48 |  |  |  |
| AC |  |  | 1.00 | — |  |
| NAC |  |  | 2.05 | 1.10, 3.82 | 0.024 |
| **Menopausal status** | 486 | 48 |  |  |  |
| Postmenopausal |  |  | 1.00 | — |  |
| Premenopausal |  |  | 0.67 | 0.38, 1.18 | 0.2 |
| **Initial clinical tumor size** | 486 | 48 |  |  |  |
| T2 |  |  | 1.00 | — |  |
| T3 |  |  | 1.23 | 0.62, 2.44 | 0.5 |
| **Initial nodal status** | 486 | 48 |  |  |  |
| N0 |  |  | 1.00 | — |  |
| N+ |  |  | 1.07 | 0.57, 2.00 | 0.8 |
| **Surgery** | 486 | 48 |  |  |  |
| Mastectomy |  |  | 1.00 | — |  |
| Lumpectomy |  |  | 0.60 | 0.33, 1.11 | 0.10 |

| **Distant Recurrence Free Survival** | **N** | **Event N** | **HR** | **95% CI** | **p-value** |
| --- | --- | --- | --- | --- | --- |
| **Chemotherapy** | 486 | 90 |  |  |  |
| AC |  |  | 1.00 | — |  |
| NAC |  |  | 1.41 | 0.88, 2.26 | 0.2 |
| **Menopausal status** | 486 | 90 |  |  |  |
| Postmenopausal |  |  | 1.00 | — |  |
| Premenopausal |  |  | 1.00 | 0.66, 1.52 | >0.9 |
| **Initial clinical tumor size** | 486 | 90 |  |  |  |
| T2 |  |  | 1.00 | — |  |
| T3 |  |  | 1.13 | 0.68, 1.87 | 0.6 |
| **Initial nodal status** | 486 | 90 |  |  |  |
| N0 |  |  | 1.00 | — |  |
| N+ |  |  | 1.46 | 0.93, 2.28 | 0.10 |
| **Surgery** | 486 | 90 |  |  |  |
| Mastectomy |  |  | 1.00 | — |  |
| Lumpectomy |  |  | 0.45 | 0.28, 0.71 | <0.001 |

| **Invasive Disease Free Survival** | **N** | **Event N** | **HR** | **95% CI** | **p-value** |
| --- | --- | --- | --- | --- | --- |
| **Chemotherapy** | 486 | 106 |  |  |  |
| AC |  |  | 1.00 | — |  |
| NAC |  |  | 1.72 | 1.12, 2.65 | 0.013 |
| **Menopausal status** | 486 | 106 |  |  |  |
| Postmenopausal |  |  | 1.00 | — |  |
| Premenopausal |  |  | 0.91 | 0.62, 1.34 | 0.6 |
| **Initial clinical tumor size** | 486 | 106 |  |  |  |
| T2 |  |  | 1.00 | — |  |
| T3 |  |  | 0.87 | 0.53, 1.42 | 0.6 |
| **Initial nodal status** | 486 | 106 |  |  |  |
| N0 |  |  | 1.00 | — |  |
| N+ |  |  | 1.27 | 0.83, 1.93 | 0.3 |
| **Surgery** | 486 | 106 |  |  |  |
| Mastectomy |  |  | 1.00 | — |  |
| Lumpectomy |  |  | 0.47 | 0.31, 0.71 | <0.001 |
|  |  |  |  |  |  |

| **Loco-Regional Free Survival** | **N** | **Event N** | **HR** | **95% CI** | **p-value** |
| --- | --- | --- | --- | --- | --- |
| **Chemotherapy** | 486 | 19 |  |  |  |
| AC |  |  | 1.00 | — |  |
| NAC |  |  | 4.23 | 1.57, 11.4 | 0.004 |
| **Menopausal status** | 486 | 19 |  |  |  |
| Postmenopausal |  |  | 1.00 | — |  |
| Premenopausal |  |  | 0.89 | 0.36, 2.20 | 0.8 |
| **Initial clinical tumor size** | 486 | 19 |  |  |  |
| T2 |  |  | 1.00 | — |  |
| T3 |  |  | 0.27 | 0.06, 1.25 | 0.094 |
| **Initial nodal status** | 486 | 19 |  |  |  |
| N0 |  |  | 1.00 | — |  |
| N+ |  |  | 1.54 | 0.59, 3.97 | 0.4 |
| **Surgery** | 486 | 19 |  |  |  |
| Mastectomy |  |  | 1.00 | — |  |
| Lumpectomy |  |  | 0.45 | 0.17, 1.19 | 0.11 |

Figure S2 - Initial distribution of the propensity score

*The x-axis represents the propensity score, which estimates the probability of receiving neoadjuvant chemotherapy (NAC) versus adjuvant chemotherapy (AC) based on baseline covariates.*

*The y-axis indicates the density of patients at each propensity score level.*

*The overlapping density curves illustrate the initial distribution of patients in the NAC and AC groups before any matching or adjustment, highlighting differences in baseline characteristics between treatment groups.*
